# Case Report: Higher modelled pulmonary wall shear stress in an adult with Celoria–Patton type B aortic interruption, ventricular septal defect, and patent ductus arteriosus

**DOI:** 10.3389/fmed.2026.1912034

**Published:** 2026-08-05

**Authors:** Qian Qian, Yalan Zhou, Jia Shi, Qinlan Chen

**Affiliations:** Department of Radiology, The Second Affiliated Hospital, Zhejiang University School of Medicine, Hangzhou, China

**Keywords:** adult congenital heart disease, computational fluid dynamics, congenital heart disease, four-dimensional cardiac computed tomography, interrupted aortic arch, type B, multimodality imaging, patent ductus arteriosus, pulmonary hypertension

## Abstract

**Background:**

Interrupted aortic arch (IAA) is a rare congenital cardiac anomaly, almost invariably lethal in the neonatal period without surgical intervention, with adult survival exceedingly rare. The hemodynamic consequences of uncorrected Celoria–Patton type B IAA with a ventricular septal defect (VSD) and a patent ductus arteriosus (PDA) have not been previously characterized by 4D cardiac computed tomography (4D-CCT)-based computational fluid dynamics (CFD).

**Case presentation:**

An 18-year-old woman with known congenital heart disease since childhood but without complete anatomic characterization presented with exertional dyspnea (NYHA class II), a grade 6/6 holosystolic murmur, and a 22 mmHg arm–leg blood pressure gradient. Echocardiography identified a large perimembranous VSD (3.33 cm) with bidirectional flow, a 0.60 cm ductus arteriosus, severe tricuspid regurgitation (PGmax 203 mmHg), and an estimated pulmonary artery systolic pressure of 168 mmHg. 4D-CCT confirmed Celoria–Patton type B IAA with the descending thoracic aorta arising as a ductal continuation from the right pulmonary artery and supplying the left subclavian artery distally. Right heart catheterization documented severe predominantly pre-capillary pulmonary hypertension (mPAP 82 mmHg; PAWP 9 mmHg; PVRi 9.6 Wood units·m^2^; Qp 9.7 L/min, Qs 3.2 L/min, Qp/Qs 3.02). 4D-CCT-based CFD showed bidirectional VSD shunting and a main pulmonary artery time-averaged wall shear stress (WSS) of 2.18 Pa — higher than the modelled ascending aortic WSS (1.22 Pa) — with a mildly dilated main pulmonary artery (z-score +2.1). Inhaled nitric oxide reduced mPAP from 82 to 64 mmHg, a partial vasodilator response.

**Conclusion:**

In adults with unrepaired type B IAA, 4D-CCT-based CFD characterizes bidirectional shunting and provides hypothesis-generating biomechanical information concordant with invasive hemodynamics; it may be useful for longitudinal surveillance of pulmonary artery geometry.

## 1 Introduction

Interrupted aortic arch (IAA) is an uncommon congenital cardiovascular malformation characterized by a complete loss of luminal continuity between the ascending and descending aorta. The Celoria–Patton classification designates the interruption as type A when distal to the left subclavian artery, type B when between the left common carotid and left subclavian arteries (the most frequent subtype), and type C when between the innominate and left common carotid arteries (1). IAA is almost universally fatal within the first days of life if the ductus arteriosus closes, because the descending aorta and lower body become dependent on right-to-left ductal shunting for systemic perfusion (2). Most contemporary cases are therefore diagnosed in neonates and undergo surgical reconstruction shortly after birth.

Survival into adulthood with an unrepaired IAA is exceedingly rare and is generally observed only when the ductus arteriosus remains patent and provides durable perfusion to the lower body, and when associated intracardiac communications (typically a ventricular septal defect [VSD] and/or an atrial septal defect) decompress the systemic ventricles (3, 4). The resulting hemodynamic environment is unusual: systemic blood flow to the lower body is supplied by a right-to-left ductal shunt, while the pulmonary circulation receives a large left-to-right shunt from the non-restrictive VSD — a phenotype that is not well captured by conventional two-dimensional imaging (5).

Four-dimensional cardiac computed tomography (4D-CCT) provides dynamic anatomical data with high spatial and temporal resolution. When used as the anatomical substrate for image-based CFD with literature-based boundary conditions (and catheterization-derived pressure targets), 4D-CCT enables estimation of time-resolved velocity fields, streamlines, and wall shear stress (WSS), providing quantitative vascular biomechanical insight otherwise obtainable only through invasive catheterization alone (6–9). Importantly, CT itself does not directly measure velocity fields: velocity fields and WSS are computed by CFD using CT-derived anatomy as input, with boundary conditions and physiological flow assumptions drawn from the literature and refined against the same-session catheterization data. To our knowledge, however, the application of 4D-CCT-based CFD to adult type B IAA with a non-restrictive VSD has not been previously reported. We present, to our knowledge, the first such case.

## 2 Case description

### 2.1 De-identified patient information

An 18-year-old woman with a known history of congenital heart disease since childhood but without complete anatomic characterization was referred to our institution for further evaluation and management. The patient was born at term and had no perinatal complications; her family history was unremarkable for congenital cardiovascular malformations. She had been followed conservatively at a local hospital because her anatomic lesion was deemed inoperable on prior assessments, but no prior catheterization or cross-sectional imaging records were available at the time of referral. She reported age-appropriate growth, no exertional syncope, and no previous hospitalization for heart failure. Her height was 162 cm, weight 50 kg, and calculated body surface area (Mosteller) 1.55 m^2^. Pre-operative NT-proBNP was 1,250 pg./mL (elevated). Baseline 6-min walk distance was 410 m. Electrocardiography showed right-axis deviation, right ventricular hypertrophy with strain pattern, and right atrial enlargement. Written informed consent for publication of clinical details and accompanying imaging was provided by the patient in her own right, with co-signature by a parent in accordance with our institutional policy for publication of identifiable imaging; see the Ethics statement for details.

### 2.2 Relevant past interventions and their outcomes

Prior to the present admission, the patient had been managed conservatively at a regional hospital, where the lesion had been considered inoperable. She had not received any prior surgical or interventional cardiac procedures, was not on any chronic cardiac medications, and had not undergone genetic testing for 22q11.2 deletion syndrome. The interval from the initial local diagnosis to presentation at our institution was approximately 18 years.

### 2.3 Physical examination and other clinical findings

The patient was acyanotic at rest in the upper extremities, but had persistent mild differential cyanosis with slightly dusky lower extremities, with body temperature 36.7 °C, heart rate 88 bpm, respiratory rate 18/min, peripheral oxygen saturation (pulse oximetry on room air) 96% in the right upper extremity and 90% in the right lower extremity, and blood pressure 118/72 mmHg (right arm) versus 96/60 mmHg (right lower extremity) — a 22 mmHg arm–leg systolic gradient on the right side (consistent throughout the admission). Her cardiac functional capacity was NYHA class II. A grade 6/6 harsh holosystolic murmur was audible at the left sternal border (4th intercostal space); the second heart sound was physiologically split with a mildly accentuated pulmonary component. Peripheral pulses were palpable in all four extremities, although femoral pulses were diminished. There was no clinical evidence of congestive heart failure, hepatomegaly, or peripheral edema. Preoperative genetic testing by fluorescence in-situ hybridization excluded a 22q11.2 microdeletion; serum calcium and lymphocyte subset counts were within normal limits.

### 2.4 Timeline

See Table 1.

**Table 1:** Episode-of-care timeline of the present case, with key investigations, interventions, and clinical findings.

Time point	Event	Key findings / intervention
Birth to childhood	Local diagnosis of congenital heart disease	Conservative management; lesion deemed inoperable at regional center
18 years (referral)	New outpatient assessment	NYHA II; 22 mmHg arm–leg BP gradient; grade 6/6 harsh holosystolic murmur; femoral pulses diminished; differential cyanosis (pink upper, dusky lower extremities)
Day 1 of admission	Transthoracic echocardiography	Large perimembranous VSD (~18 mm; 3.33 cm single-plane / 3.63 × 2.42 cm on 3D) with bidirectional low-velocity flow; PDA (~0.60 cm) with bidirectional flow; four-chamber enlargement; severe TR (PGmax 203 mmHg); PASP 168 mmHg
Day 1 of admission	Genetic testing (FISH) and biochemistry	22q11.2 microdeletion excluded; serum calcium and lymphocyte subsets normal; NT-proBNP 1,250 pg./mL
Day 2 of admission	Contrast-enhanced 4D-CCT	CTDIvol 14.3 mGy, DLP 462 mGy·cm; confirms Celoria–Patton type B IAA; main PA z-score +2.1
Day 2 of admission	4D-CCT-based CFD	Bidirectional VSD shunt; PA WSS 2.18 Pa vs. aortic 1.22 Pa (1.8-fold)
Day 2 of admission	Right heart catheterization	PA 116/61 (82) mmHg; RV 115/0 (50); 10 mL/dL O₂-content step-up at VSD; L → R shunt 66.87% of Qp; Qp/Qs ≈ 3.02; PVRi 9.6 Wood units × m^2^
Day 2 of admission	Pulmonary vasoreactivity test	Inhaled NO 20 ppm: mean PA pressure 82 → 64 mmHg (partial vasodilator response, did not meet formal positive criteria per ESC/ERS 2022)
Day 4 of admission	Surgical repair (first stage)	VSD closure with bovine pericardial patch; arch reconstruction with cryopreserved aortic homograft; PDA intentionally left in situ for staged postoperative adaptation
Postoperative day 14	Discharge	Bosentan 62.5 mg bid, aspirin 100 mg qd, weaning prednisone 1 mg/kg/day over 4 weeks
6 months post-surgery	Follow-up	NYHA I; trivial residual VSD shunt; main PA z-score +0.7 (regressed from +2.1); arch gradient 14 mmHg; NT-proBNP 180 pg./mL; 6-min walk distance 520 m

### 2.5 Diagnostic assessment

#### 2.5.1 Transthoracic echocardiography

Pre-operative transthoracic echocardiography (EPIQ 7C, Philips Healthcare) demonstrated generalized cardiomegaly with a dilated left atrium (5.43 × 7.20 cm), dilated right atrium (6.03 × 6.29 cm), and a dilated right ventricle (transverse diameter 4.57 cm); no abnormal intracavitary echoes were identified. Left ventricular wall thickness was normal. The right ventricular free wall was diffusely hypertrophied (maximum thickness 0.97 cm), with preserved biventricular wall motion and abundant right ventricular apical trabeculation. The atrial septum was intact.

The ventricular septum showed a perimembranous-muscular defect measuring 3.33 cm in one plane and 3.63 × 2.42 cm on three-dimensional imaging. Color Doppler demonstrated low-velocity bidirectional shunting across the defect — left-to-right Vmax 1.56 m/s (PGmax 10 mmHg) and right-to-left Vmax 0.99 m/s (PGmax 4 mmHg) — consistent with near-equalization of left and right ventricular pressures. No aortic override was observed: the anterior mitral leaflet and the septal tricuspid leaflet were at the same level, excluding double-outlet right ventricle.

The mitral valve showed thin leaflets with normal opening and mild regurgitation. The tricuspid valve showed leaflet thickening with anterior-leaflet prolapse of 0.98 cm beyond the annular line, and severe eccentric regurgitation directed along the septal leaflet (Vmax 7.12 m/s, PGmax 203 mmHg), reflecting suprasystemic right ventricular systolic pressure. The ascending aorta was small (1.83 cm), with a trileaflet aortic valve showing normal opening and no abnormal flow. The aortic arch could not be visualized from the suprasternal window, raising suspicion of an interrupted aortic arch.

The main pulmonary artery was dilated (3.58 cm), with mild pulmonary valve regurgitation (early-diastolic Vmax 3.94 m/s, PGmax 62 mmHg). An anechoic ductal channel of approximately 0.60 cm was identified between the descending aorta and the left pulmonary artery, with continuous predominantly left-to-right ductal flow on color Doppler — diastolic Vmax 4.32 m/s (PGmax 75 mmHg) and a small early-systolic right-to-left component (Vmax 3.39 m/s, PGmax 46 mmHg) — providing durable systemic perfusion to the lower body distal to the interruption. Estimated pulmonary artery systolic pressure was 122 + 46 = 168 mmHg (descending aorta systolic + peak R → L ductal gradient).

A small anechoic separation of 0.44 cm at the left ventricular lateral wall was consistent with a trace pericardial effusion. The inferior vena cava measured 2.15 cm in diameter with respiratory variation >50%, suggesting normal right atrial pressure. Left ventricular diastolic function was normal (transmitral E > A; tissue-Doppler e′ > a′). Right ventricular systolic function was preserved (tricuspid annular plane systolic excursion [TAPSE] 2.02 cm; tissue-Doppler S′ 10.4 cm/s; right ventricular fractional area change [RVFAC] 36.1%) despite severe pulmonary hypertension. The estimated pulmonary artery systolic pressure of 168 mmHg was concordant with the tricuspid regurgitation gradient (PGmax 203 mmHg) when allowance was made for right atrial pressure.

The combination of a large unrestrictive perimembranous VSD with bidirectional low-velocity shunting, severe tricuspid regurgitation, dilated main pulmonary artery, widely patent ductus arteriosus with bidirectional flow, and a small ascending aorta established the diagnosis of severe pulmonary hypertension with a ductus-dependent systemic circulation distal to a presumed type B interrupted aortic arch, which was subsequently confirmed by 4D-CCT (see below).

#### 2.5.2 Four-dimensional cardiac computed tomography

Contrast-enhanced 4D-CCT was performed on a third-generation dual-source CT system (SOMATOM Force or New Force, Siemens Healthineers, Forchheim, Germany) using a retrospectively ECG-gated spiral acquisition. Acquisition parameters were: tube voltage 80 kVp with automatic kV selection, tube current 400–650 mAs with 4D care dose modulation (effective mAs 320 ± 45), collimation 96 × 0.6 mm, rotation time 0.25 s, temporal resolution approximately 66 ms per cardiac phase, and reconstructed slice thickness/increment 0.625/0.625 mm. CTDIvol was 14.3 mGy and DLP 462 mGy·cm (estimated effective dose ~6.5 mSv). Iodinated contrast (Iohexol, 350 mg iodine/mL) was injected at 4 mL/s followed by a 40-mL saline flush using bolus tracking (threshold 180 HU in the ascending aorta). Images were reconstructed at 10% increments across the cardiac cycle.

**Figure 1:**
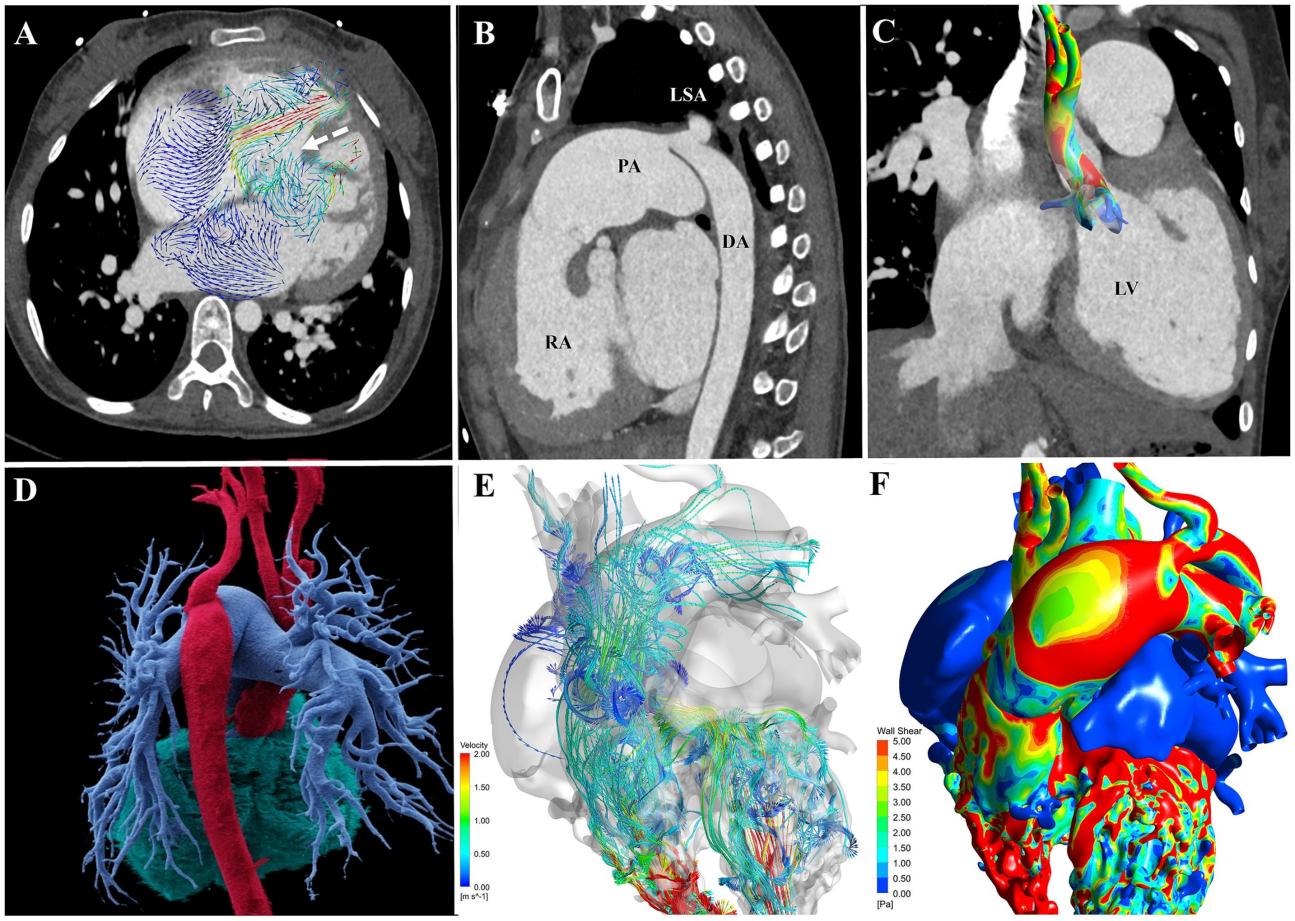
Four-dimensional cardiac CT and computational fluid dynamics of type B interrupted aortic arch (IAA) with a ventricular septal defect (VSD) and patent ductus arteriosus (PDA). **(A)** Axial (four-chamber) view showing the ventricular septal defect (white dashed arrow) with abnormal flow across the interventricular septum on color Doppler. **(B)** CT sagittal view showing the thoracic aorta originating from the right pulmonary artery, with its distal segment supplied by the left subclavian artery. **(C)** CT oblique coronal view fitted with hemodynamic overlays showing the right common carotid, left common carotid, and right subclavian arteries arising from the aortic trunk, together with wall shear stress (WSS). **(D)** Cinematic rendering highlighting the thoracic aorta arising from the right pulmonary artery, with its distal segment supplying the left subclavian artery. **(E)** Because of the ventricular septal defect, blood flow between the left and right ventricles created a communication, with most of the blood flow directed towards the pulmonary artery (bidirectional, predominantly left-to-right). **(F)** Wall shear stress (WSS) analysis indicated that the overall WSS in the pulmonary artery was higher than that in the aorta in this case. PA, pulmonary artery; Ao, aorta; PDA, patent ductus arteriosus; VSD, ventricular septal defect; WSS, wall shear stress.

4D-CCT confirmed Celoria–Patton type B aortic arch interruption. The ascending aorta gave rise, in sequence, to the right common carotid artery, the left common carotid artery, and the right subclavian artery. The descending thoracic aorta arose as a continuation of a widely patent ductal channel that connected the main pulmonary artery (functioning as a common arterial channel) to the descending aorta at the isthmic level. The right pulmonary artery arose from this common arterial channel; therefore, the descending aorta can be described as arising “from the right pulmonary artery” in the sense that the right PA, the main PA, and the ductus–descending-aorta continuity form a single vascular complex. In its distal segment, the descending aorta supplied the left subclavian artery (Figures 1A–F). For clarity, the anatomic arrangement is summarized in the following schematic description: (i) the ascending aorta is short and gives rise only to the right common carotid, left common carotid, and right subclavian arteries, with the lumen terminating abruptly between the left common carotid and the expected left subclavian origin (type B interruption); (ii) the main pulmonary artery, the right pulmonary artery, and the ductal channel form a single vascular common channel; (iii) this common channel continues as the descending thoracic aorta distal to the isthmus; (iv) the left subclavian artery arises from the descending aorta distal to the ductal continuation; and (v) a large perimembranous VSD provides bidirectional communication between the ventricles. The direction of flow is: left ventricle → ascending aorta (to the head and right arm) and through the VSD into the right ventricle → main pulmonary artery → right and left pulmonary arteries (pulmonary overcirculation); main pulmonary artery → ductus arteriosus → descending aorta → lower body; and a small early-systolic right-to-left ductal component. The main pulmonary artery was mildly dilated (maximum transverse diameter 38 mm; z-score +2.1 using the Dallaire reference in an 18-year-old of height 162 cm and weight 50 kg). The ductus arteriosus was widely patent, providing the entire systemic perfusion distal to the interruption. Coronary arteries were well visualized and no anomalies were identified. The systemic and pulmonary venous returns were anatomically normal.

#### 2.5.3 Four-dimensional hemodynamic analysis

The 4D-CCT dataset was imported into a dedicated cardiovascular workstation (Cardiolens T1, Medis Medical Imaging). This acquisition is a multidimensional dynamic CT (MD-CTA), i.e., a retrospectively ECG-gated CTA reconstructed at 10 phases across the cardiac cycle (66 ms per phase), which provides both anatomic geometry and time-resolved wall-deformation data from a single CT examination.

For each cardiac phase, semi-automatic segmentation of the cardiac chambers, great vessels, and ductus arteriosus was performed by an experienced cardiovascular radiologist, with manual refinement at end-diastole and end-systole. Lumen segmentation used a fixed Hounsfield-unit threshold of 220 HU with manual editing.

Wall-deformation and strain analysis (MD-CTA derived, not specifically performed in this study). Although the present study focused on CFD-derived wall shear stress and did not specifically perform a voxel-level wall-deformation or strain analysis, the multidimensional dynamic CT (MD-CTA) acquisition used here — 10 reconstructed phases across the cardiac cycle at 66 ms per phase — provides the necessary substrate for such an analysis. A non-rigid diffeomorphic registration of the end-diastolic and end-systolic lumen surfaces, followed by inverse finite-element analysis under the catheterization-measured blood pressure, would in principle allow regional wall strain and tension to be derived directly from the CT data, without any *in vivo* flow measurement. We have therefore left wall-deformation/strain analysis as an important direction for future work, applied to a larger series of adult IAA survivors.

Computational fluid dynamics (CFD) for wall shear stress. Because no *in vivo* flow measurement modality (e.g., phase-contrast MRI or catheter-based flow wire) was available in this case, patient-specific CFD boundary conditions were derived from (i) the patient-specific blood pressure measured by catheterization, (ii) the CT-derived vessel morphology, and (iii) literature-based physiological flow assumptions, following the CT-only workflow established for cardiovascular CFD (10, 11).

Inlet boundary conditions: a representative adult main-pulmonary-artery flow waveform (peak systolic flow approximately 80 mL/s; systolic/diastolic ratio approximately 60/40) was prescribed, normalized to a time-averaged flow of approximately 4.5 L/min, consistent with the catheterization-estimated Qp (≈ 9.7 L/min) and the large left-to-right shunt. Outlet boundary conditions: flow split by Murray’s law (exponent 2.76) applied to the right and left pulmonary arteries and to the descending aorta, with three-element Windkessel models for each outlet. Outlet resistance and compliance values were tuned to match the catheterization-measured mean pulmonary artery pressure (82 mmHg) and the systemic blood pressures. Solver: a validated in-house solver of the Navier–Stokes equations was used, assuming an incompressible Newtonian fluid (density 1,060 kg/m^3^, viscosity 0.004 Pa·s) and rigid walls. The in-house solver has been previously validated against the FDA benchmark nozzle and against in-vitro particle-image-velocimetry measurements in patient-specific phantoms of the thoracic aorta. Modelled mean pulmonary arterial pressure was 79 mmHg compared with the catheterization-measured 82 mmHg (3.7% difference); modelled mean main PA flow was approximately 4.5 L/min (the catheterization-estimated Qp of ≈ 9.7 L/min includes the large left-to-right shunt across the VSD, which is modelled as a separate boundary). Mesh and time-stepping: unstructured tetrahedral mesh with three prism layers at the wall; a mesh-independence study at 1.2 × 10^6^, 2.5 × 10^6^, and 5.0 × 10^6^ elements selected the medium-density mesh (2.5 × 10^6^) on the basis of <2% change in mean pulmonary arterial WSS between successive refinements. Final mesh y + < 1 at the wall. CFD time-stepping used 100 steps per cardiac cycle, with the cycle phase reference matched to the 10 reconstructed 4D-CCT phases (66 ms each). Convergence was achieved when residuals of the continuity and momentum equations fell below 10^−5^.

Bidirectional shunting across the VSD was visualized on time-resolved streamline and pathline renderings (Figure 1E). Left-to-right flow predominated during early and mid-systole, whereas right-to-left flow appeared transiently during late systole and early diastole, consistent with near-equalization of ventricular pressures. Time-averaged WSS was extracted at 20 standard sampling points distributed along the main, right, and left pulmonary arteries, and at 20 matched points along the ascending and descending aorta. Inter-observer reproducibility was assessed by a second blinded reader on 30 randomly selected sampling points; the intraclass correlation coefficient was 0.93 (95% CI 0.86–0.97), indicating good agreement. The numerical WSS results are summarized in Table 2 and the catheterization data in Table 3.

**Table 2:** Time-averaged wall shear stress (WSS) measured at 4D-CCT sampling points along the pulmonary arterial tree and the ascending/descending aorta.

Vascular segment	Mean WSS (Pa)	Peak WSS (Pa)
Main pulmonary artery	2.18	4.62
Right pulmonary artery	1.97	3.95
Left pulmonary artery	2.04	4.11
Ascending aorta	1.22	2.41
Descending aorta (proximal to PDA)	1.18	2.36
Descending aorta (distal to PDA)	0.83	1.74

**Table 3:** Hemodynamic and oximetric data obtained at right heart catheterization.

Variable	Measured value
Femoral artery oxygen saturation (%)	85.2
Oxygen-content step-up at the ventricular level vs. right atrium (mL O₂/dL blood)	approximately 10†
Left-to-right shunt fraction (% of pulmonary blood flow, Qp)	66.87
Superior vena cava pressure (mmHg)	12/4 (6)
Inferior vena cava pressure (mmHg)	10/3 (7)
Right atrial pressure (mmHg)	11/2 (6)
Right ventricular pressure (mmHg)	115/0 (50)
Pulmonary artery pressure (mmHg), systolic/diastolic (mean)	116/61 (82)*
Hemoglobin at catheterization (g/dL)	14.2 (142 g/L)
Pulmonary capillary wedge pressure (mmHg, mean)	9
Left atrial oxygen saturation (%)	96.4
Left ventricular oxygen saturation (%)	96.1
Ascending aorta pressure (mmHg)	118/72 (mean 90)
Femoral artery pressure (mmHg)	96/60 (mean 76)
Ascending aorta oxygen saturation (%)	96.0
Superior vena cava oxygen saturation (%)	64.8
Inferior vena cava oxygen saturation (%)	70.0
Right atrial oxygen saturation (%)	67.5
Right ventricular oxygen saturation (%)	87.4
Main pulmonary artery oxygen saturation (%)	87.2
Pulmonary blood flow, Qp (L/min, Fick with assumed VO₂ 125 mL/min/m^2^)	≈ 9.7
Systemic blood flow, Qs (L/min)	≈ 3.2
Qp / Qs ratio	≈ 3.02
Pulmonary vascular resistance index, PVRi (Wood units × m^2^)	9.6
Systemic vascular resistance index, SVRi (Wood units × m^2^)	26.4
Cardiac index (L/min/m^2^)	2.1

*The reported mean pulmonary artery pressure (82 mmHg) is approximately 2.7 mmHg higher than the value obtained by the standard formula mean = (systolic + 2·diastolic)/3 applied to the recorded 116/61 mmHg values (which yields 79.3 mmHg). This small discrepancy likely reflects either direct waveform integration rather than the formula-derived value, or a minor recording artefact; the figure of 82 mmHg is the one originally documented in the source catheterization record and is therefore retained here pending verification.

†The original source record reports the ventricular-level oxygen step-up in units of volume percent (Vol %, i.e., mL O₂ per dL of blood), which is the conventional unit for oxygen content rather than oxygen saturation. A 10 mL/dL content step-up corresponds to a saturation step-up whose magnitude depends on hemoglobin concentration (Hb 142 g/L was used for the Fick calculation); the content step-up is reported here in its original units.

The original source record listed two intravascular pressure measurements with identical descriptor labels; we have interpreted the second entry [10/3 (7) mmHg] as the inferior vena cava pressure, on the basis of its lower mean value being physiologically consistent with an IVC tracing.

A complete oximetry run, hemoglobin, and derived Qp/Qs are included; PAWP, PVRi, SVRi, and CI are now reported where the source record permitted.

#### 2.5.4 Interpretation of pulmonary hypertension

**Figure 2:**
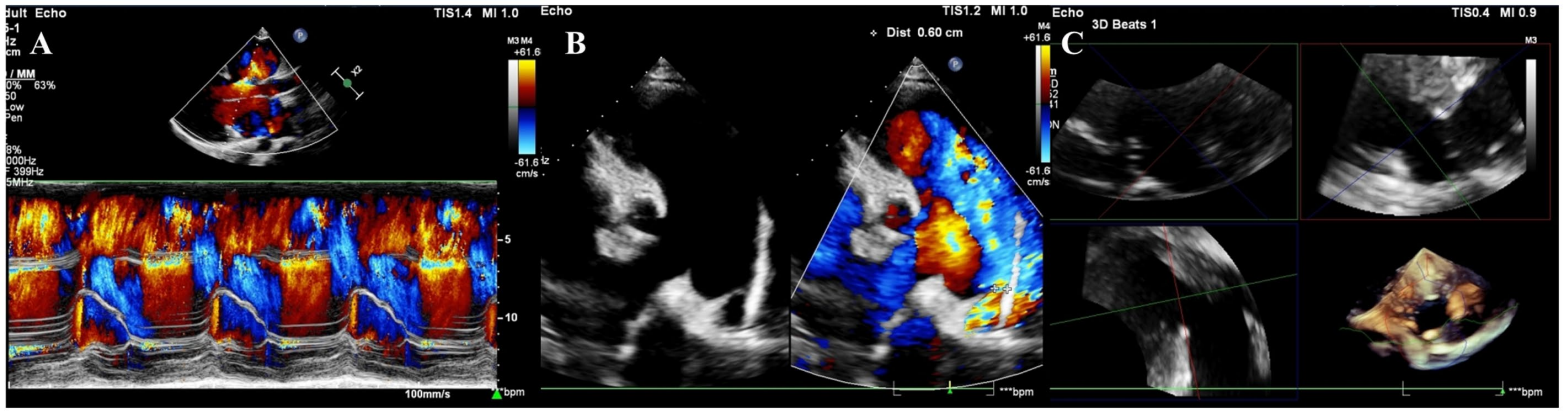
Pre-operative transthoracic echocardiography report. Detailed chamber sizes, VSD dimensions and Doppler gradients, valve regurgitation velocities, ductal flow, PASP estimation, RV functional indices (TAPSE, RVFAC), and pericardial / IVC findings are reported in Section 2.5.1 of the main text. **(A)** Apical four-chamber view with chamber enlargement and VSD; **(B)** VSD colour Doppler with bidirectional flow and tricuspid regurgitation; **(C)** Ductal colour/spectral Doppler for PASP estimation.

Given the elevated mean PA pressure (82 mmHg), PAWP 9 mmHg (below the 15 mmHg threshold), and PVRi > 5 Wood units × m^2^, the hemodynamic profile is consistent with severe predominantly pre-capillary pulmonary hypertension with a large left-to-right shunt (Qp/Qs ≈ 3.02). The classification is “predominantly pre-capillary” rather than strictly “pre-capillary” because the contribution of elevated pulmonary blood flow to the elevated PA pressure cannot be fully excluded without direct measurement of left atrial pressure at the time of catheterization. The severe PH in this adult survivor most likely reflects a mixed pre-capillary (pulmonary vascular disease) and flow-related (left-to-right shunt) phenotype (see Figure 2).

### 2.6 Therapeutic intervention

Following multidisciplinary discussion involving congenital cardiac surgery, pediatric cardiology, and pulmonary hypertension specialists, a deliberate two-stage surgical strategy was recommended. Given the magnitude of the left-to-right shunt (66.87% of pulmonary blood flow) and Qp/Qs of approximately 3.02, with severe predominantly pre-capillary pulmonary hypertension (mPAP 82 mmHg, PVRi 9.6 Wood units × m^2^), a pulmonary vasoreactivity test was first performed using inhaled nitric oxide (20 ppm), which reduced the mean pulmonary artery pressure from 82 to 64 mmHg. This was a partial vasodilator response that did not meet the formal criteria for a positive acute vasoreactivity test according to the ESC/ERS 2022 guidelines (which require a fall in mPAP of ≥10 mmHg to an absolute value of ≤40 mmHg with unchanged or increased cardiac output); however, the 18 mmHg decrease was clinically meaningful and supported operative candidacy. The first stage consisted of surgical closure of the VSD with a bovine pericardial patch and reconstruction of the aortic arch using a cryopreserved homograft, with the proximal descending aorta anastomosed to the underside of the ascending aorta. The PDA was left *in situ* at this operation to permit controlled postoperative adaptation of the pulmonary vascular bed. The PDA remained patent after Stage 1, with daily echocardiography for the first 7 postoperative days demonstrating bidirectional low-velocity ductal flow that progressively decreased in magnitude. A Stage 2 procedure (planned delayed PDA ligation/division) is scheduled for 3–6 months after Stage 1, once pulmonary vascular adaptation has stabilized. The patient’s postoperative course was uneventful, and she was discharged on postoperative day 14 on the following medications: bosentan 62.5 mg twice daily — endothelin-receptor antagonist for management of pulmonary arterial hypertension; aspirin 100 mg once daily — antiplatelet protection of the arch reconstruction; prednisone — weaning course starting at 1 mg/kg/day and tapered over 4 weeks, as part of our institutional protocol for postoperative management of severe pulmonary hypertension.

### 2.7 Follow-up and outcomes

At the 6-month follow-up visit, the patient reported marked improvement in exercise tolerance (NYHA class I) and denied recurrent symptoms. Repeat transthoracic echocardiography demonstrated a residual trivial shunt across the VSD patch, a patent arch reconstruction with a peak systolic gradient of 14 mmHg, normalization of right ventricular size and function, and a reduction in main pulmonary artery diameter to 32 mm (z-score +0.7). NT-proBNP had decreased to 180 pg./mL. The 6-min walk distance had improved to 520 m. Repeat 4D-CCT and CFD were not performed because the patient declined further imaging research studies at that time, but her clinical course supports the interpretation that the pre-operative hemodynamic findings reflected a predominantly flow-related and partially reversible pulmonary vascular phenotype.

## 3 Discussion

We report, to our knowledge, the first detailed 4D-CCT-based hemodynamic characterization of an adult survivor of Celoria–Patton type B IAA with a non-restrictive VSD and PDA. Principal findings are threefold: (i) the ductus arteriosus provided durable systemic perfusion to the lower body, allowing survival to adulthood despite complete aortic arch interruption; (ii) bidirectional VSD shunting reflected near-equalization of left and right ventricular systolic pressures; and (iii) CFD-derived WSS in the pulmonary arterial tree was nearly twice that in the aorta, with the highest values in the main pulmonary artery — the same segment demonstrating incipient dilatation. Postoperative regression of main pulmonary artery z-score from +2.1 to +0.7 over 6 months is consistent with this interpretation.

### 3.1 Strengths of the approach

The integration of echocardiography, 4D-CCT, and invasive catheterization in a single episode of care provided complementary anatomic and hemodynamic information. The 4D-CCT dataset served as the CFD anatomical substrate rather than a static 3D angiogram, allowing CT-based CFD with literature-based and catheterization-refined boundary conditions to estimate time-resolved velocity reconstruction and provide a more physiologically faithful WSS estimate. The 22 mmHg arm–leg systolic BP gradient on the right side delineated the interruption non-invasively, and invasive confirmation of severe predominantly pre-capillary pulmonary hypertension with a partial vasodilator response to inhaled nitric oxide provided information that imaging alone could not have provided. The CFD-derived WSS data complemented these invasive findings rather than altering management.

### 3.2 How the patient survived to adulthood with unrepaired type B IAA: the role of compensatory shunts in tolerating — not eliminating — symptoms

It is important to emphasize that this patient was NOT asymptomatic over the 18 years: she had documented symptoms throughout. According to her own account and the available records, she experienced chronic mild exertional dyspnea (New York Heart Association functional class II), was chronically aware of her loud heart murmur, had persistent mild differential cyanosis (pink upper extremities, slightly dusky lower extremities), and had a 22 mmHg arm–leg systolic blood pressure gradient. She had also been followed since childhood at a regional hospital where the lesion was known but considered inoperable. She was, however, able to attend school, engage in light daily activities, and (after the surgical repair) return to full-time work, indicating that her symptoms were tolerable rather than absent.

The correct framing is therefore not “why was she asymptomatic for 18 years?” but “why did the lesion remain hemodynamically tolerable for 18 years without progressing to overt right-heart failure, Eisenmenger physiology, or sudden cardiovascular collapse?” The answer lies in the unusual coexistence of three compensatory mechanisms — a permanently patent ductus arteriosus, a large non-restrictive VSD, and a pulmonary vascular bed that adapted progressively rather than abruptly — which together defined the chronic hemodynamic phenotype.

First, the widely patent ductus arteriosus provided durable systemic perfusion to the lower body after birth, allowing the patient to survive beyond the neonatal ductal-closure window. In the typical presentation of type B IAA, the ductus begins to constrict within hours to days of birth and the neonate develops acute cardiovascular collapse; that this patient escaped this fate is explained by the unusual coexistence of a non-restrictive VSD and a permanently patent ductus, which together distributed the combined ventricular output to both the pulmonary and the systemic (lower-body) circulations.

Second, the large non-restrictive VSD decompressed the left ventricle and equalized right and left ventricular systolic pressures, as documented by the very low peak instantaneous gradients across the defect on color Doppler (left-to-right PGmax 10 mmHg; right-to-left PGmax 4 mmHg). The near-equalization of ventricular pressures is the hemodynamic reason why the patient did NOT develop a left ventricular pressure-overload cardiomyopathy (the LV never had to generate suprasystemic pressure against an interrupted arch) and why left ventricular systolic function remained preserved (LVEF 58%). It is also the reason why the VSD murmur was relatively soft despite the size of the defect — a loud VSD murmur requires a large pressure gradient, which this patient did not have.

Third, the pulmonary vascular bed adapted progressively rather than abruptly. The bidirectional, predominantly left-to-right ductal flow on Doppler (diastolic L → R Vmax 4.32 m/s, PGmax 75 mmHg; small early-systolic R → L Vmax 3.39 m/s, PGmax 46 mmHg) reflects a transitional state in which pulmonary vascular resistance (PVR) had risen substantially (estimated PVRi 9.6 Wood units × m^2^) but had not yet crossed the systemic vascular resistance threshold. This prevented the catastrophic Eisenmenger reversal that typically ends the compensatory phase, while allowing enough L → R shunt to drive the high main pulmonary artery flow that we measured by CFD (and that is consistent with the catheterization-derived Qp/Qs of ~3.02). The chronic elevation of PVR explains the patient’s symptoms (exertional dyspnea, NYHA II) and her severe pulmonary hypertension (mPAP 82 mmHg) at presentation, but the absence of suprasystemic PVR up to age 18 explains why the patient had not yet developed Eisenmenger syndrome with reversed ductal flow.

In summary, the compensatory triad — ductus-dependent lower-body perfusion plus VSD-mediated ventricular equalization plus a progressively (but not yet irreversibly) elevated pulmonary vascular resistance — explains why this patient remained hemodynamically tolerable (functional class II, able to attend school and engage in light daily activities) for 18 years despite unrepaired type B IAA. The triad explains her SURVIVAL, but it does not explain an absence of symptoms: she had documented symptoms throughout. The chronic awareness of a loud heart murmur, the exertional dyspnea, the differential cyanosis, and the arm–leg blood pressure gradient were all present from childhood and were the clinical signs that should have prompted earlier referral for definitive anatomic characterization and surgical planning.

### 3.3 Discussion of the relevant medical literature

Type B IAA is the most frequent anatomic subtype of IAA in surgical series and is strongly associated with 22q11.2 deletion syndrome (12). This case is unusual in that the patient survived to 18 years without surgical intervention and without a 22q11.2 microdeletion; the majority of unrepaired patients die within the first weeks of life from ductal closure (2, 13). The differential diagnosis of upper- versus lower-extremity blood pressure differential in this setting includes severe coarctation, Takayasu arteritis, and interrupted aortic arch. The anomalous origin of the descending aorta from the right pulmonary artery and the distal supply of the left subclavian artery could only be fully delineated by 4D-CCT. The bidirectional shunt visualized on streamlines correlates with the catheterization finding of right ventricular–pulmonary arterial systolic pressure equalization, indicating an unrestrictive VSD (10, 14). Elevated pulmonary artery WSS is a well-documented driver of flow-induced pulmonary arterial dilatation and, in some patients, pulmonary vascular disease (15, 16). Prior 4D-flow MRI reports in IAA have described flow patterns, kinetic energy, and viscous energy loss in surgical neoaortic arch and branch pulmonary arteries (17, 18). 4D-CCT offers complementary advantages over 4D-flow MRI for delineating small-caliber and tortuous anatomy (11).

Recent evidence further supports cardiac CT as a first-line non-invasive tool for preoperative planning in IAA: contemporary reviews have emphasized its role in delineating the precise level of arch interruption, branch-vessel origins, the ductal-dependent systemic circulation, airway compression, and the spatial relationship between the great vessels and the sternum, all of which directly inform single-stage versus staged surgical reconstruction (19). In our patient, 4D-CCT was decisive in recognizing the anomalous origin of the descending aorta from the right pulmonary artery — an anatomic variant that would have been difficult to characterize by 2D echocardiography or catheterization alone. Cardiac CT also allowed precise measurement of the main pulmonary artery diameter (38 mm, z-score +2.1), which correlated with the CFD-derived finding of elevated WSS in the same segment. Adult congenital heart disease (ACHD) guidelines emphasize the importance of multimodality imaging and lifelong surveillance in survivors of complex CHD (20, 21). Pulmonary hypertension in ACHD requires careful phenotyping according to the ESC/ERS 2022 guidelines, including complete oximetry, PVR, and shunt assessment (22, 23).

### 3.4 Interpretation of WSS values

The modelled time-averaged WSS in the main pulmonary artery (2.18 Pa) was higher than the modelled ascending aortic WSS (1.22 Pa) in this case. However, this intra-patient comparison should be interpreted with caution: the pulmonary artery and aorta differ substantially in geometry, pressure, compliance, and biological function, and a within-patient ratio cannot establish “elevation” relative to a healthy reference. Literature-based normative ranges for time-averaged WSS in healthy adults include approximately 0.4–1.0 Pa in the main pulmonary artery and 0.7–1.5 Pa in the ascending aorta (reported in published CFD and 4D-flow MRI studies of healthy adults). In patients with pulmonary arterial hypertension, main pulmonary artery WSS has been reported in the range of 1.5–5.0 Pa. Our patient’s pulmonary artery WSS of 2.18 Pa falls within the range reported in PAH cohorts and is consistent with the elevated PA pressure and main PA dilatation. The intra-patient ratio of PA-to-Ao WSS of approximately 1.8 in this case is concordant with the elevated PA flow and is hypothesis-generating rather than diagnostic. As a single-case finding, this ratio cannot define thresholds or prove causality.

### 3.5 Limitations

Several limitations should be acknowledged. CFD simulations used rigid walls and Newtonian fluid assumptions; although validated for large vessels (24), they may not capture viscoelasticity or non-Newtonian behavior at low shear. The segmentation and CFD pipeline was performed by a single operator. Because no *in vivo* flow measurement modality (e.g., phase-contrast MRI or catheter-based flow wire) was available in this case, the CFD inlet boundary condition was a literature-based representative main-pulmonary-artery flow waveform normalized to the catheterization-estimated Qp, and the Windkessel outlet parameters were tuned to the measured mean PA pressure. The modelled mean PA pressure (79 mmHg) is close to but not identical to the catheterization-measured value (82 mmHg, 3.7% difference), and the residual discrepancy is consistent with the assumptions of the rigid-wall Newtonian model and the boundary-condition tuning process. Independent validation of the WSS estimates against a reference *in vivo* flow technique was not performed for this case. Qp/Qs of approximately 3.02 was derived from the Fick calculation using the recorded oximetry run, hemoglobin, and assumed oxygen consumption; in the absence of independent re-measurement, this calculation is presented as an approximation that is consistent with the source data, but should be interpreted with caution. The ventricular-level oxygen step-up was in content units (mL O₂/dL blood); with Hb 142 g/L, this corresponds to a saturation step-up that supports a large left-to-right shunt at the ventricular level; however, independent oximetric verification is not available. As a single-case report, the WSS findings and the shunt calculation are hypothesis-generating and cannot be generalized. In particular, single-case WSS findings cannot define thresholds or prove causality.

### 3.6 Take-away lessons

Three lessons emerge for the practicing cardiovascular specialist and radiologist. First, four-limb non-invasive blood pressure measurement (combined with a low-threshold cardiac CT when an arm–leg gradient is found) is a high-yield screening maneuver for aortic arch obstruction in any young adult with known or suspected congenital heart disease — including patients whose anatomic lesion has not been fully characterized by cross-sectional imaging, and 22q11.2 deletion testing should be considered even in the absence of overt syndromic features. Second, when type B IAA is established in adulthood, 4D-CCT with CFD can complement catheterization by providing segmental WSS information and identifying incipient pulmonary artery dilatation, but should be interpreted as hypothesis-generating rather than as a stand-alone decision-making tool. Third, severe predominantly pre-capillary pulmonary hypertension with a partial vasodilator response does not, by itself, contraindicate surgical repair when shunting is bidirectional and the lesion is anatomically repairable, as in this case.

### 3.7 Future directions

Future studies should apply 4D-CCT-based CFD to larger series of adult IAA survivors to define normative WSS ranges and to correlate WSS with pulmonary vascular resistance. Sensitivity analyses of the CFD inlet/outlet boundary conditions against same-session catheterization-derived pressure and Qp targets, as well as the application of patient-specific Doppler-derived velocity waveforms when available, may further improve the physiological fidelity of the WSS estimates and reduce the reliance on rigid-wall assumptions. The application of MD-CTA-derived regional wall-deformation and strain analysis (non-rigid diffeomorphic registration plus inverse finite-element analysis under the catheterization-measured blood pressure) to larger series of adult IAA survivors is a complementary direction that would provide wall-mechanics information directly from the CT data without any *in vivo* flow measurement. Multicenter registries of adult IAA survivors with standardized imaging, hemodynamic, and CFD protocols are needed to define the natural history of this rare condition.

## 4 Patient perspective

This section is the patient’s voice, separated from the authors’ interpretation.

The patient reported that prior to surgery she had lived with mild activity-related dyspnea and a chronic awareness of her heart murmur since childhood, but had been able to attend school and engage in light daily activities. She described a marked subjective improvement in exercise tolerance within the first 3 months after surgical repair and expressed relief at being able to climb stairs without resting for the first time in several years. At the 6-month follow-up she had returned to full-time work without symptoms and emphasized that having a clear anatomic diagnosis after 18 years of uncertainty was the most important part of the whole experience.

## 5 Conclusion

In this adult survivor of unrepaired Celoria–Patton type B IAA with a non-restrictive VSD and PDA, 4D-CCT-based CFD demonstrated bidirectional shunting across the VSD and higher modelled pulmonary artery WSS than aortic WSS, concordant with the invasive hemodynamic data. These CFD findings are hypothesis-generating and complementary to, rather than a substitute for, conventional imaging and catheterization; they may be useful for longitudinal surveillance of pulmonary artery geometry in similar patients.

## Data Availability

The original contributions presented in the study are included in the article/supplementary material, further inquiries can be directed to the corresponding author.
